# Unexpectedly long half-lives of blood 2,3,4,7,8-pentachlorodibenzofuran (PeCDF) levels in Yusho patients

**DOI:** 10.1186/s12940-015-0059-y

**Published:** 2015-09-17

**Authors:** Shinya Matsumoto, Manabu Akahane, Yoshiyuki Kanagawa, Jumboku Kajiwara, Chikage Mitoma, Hiroshi Uchi, Masutaka Furue, Tomoaki Imamura

**Affiliations:** Department of Public Health, Health Management and Policy, Nara Medical University School of Medicine, 840 Shijo-cho, Kashihara, Nara Japan; Fukuoka Institute of Health and Environmental Sciences, 39 Mukaizano, Dazaifu, Fukuoka Japan; Department of Dermatology, Graduate School of Medical Sciences, Kyushu University, 3-1-1 Maidashi, Higashi-ku, Fukuoka Japan

**Keywords:** Yusho, 2,3,4,7,8-pentachlorodibenzofuran (PeCDF), Half-life, Dioxin, Dioxin-like compounds, Aging

## Abstract

**Background:**

Dioxins and dioxin-like compounds have half-lives typically between 7.2 years and 15 years. Our previous study of patients poisoned by extremely high concentrations of 2,3,4,7,8-pentachlorodibenzofuran (PeCDF) in the ‘Yusho incident’ in 1968 found that in some the half-life of blood 2,3,4,7,8-PeCDF tended towards infinity. This suggests that there are two groups of Yusho patients, those with 2,3,4,7,8-PeCDF half-lives around 10 years, and those with half-lives near infinity. We sought to establish the proportions of each in a cohort of 395 Yusho patients, and whether the proportions were changing over time.

**Methods:**

We undertook longitudinal measurement of the blood concentration of 2,3,4,7,8-PeCDF in our cohort between 2002 and 2010. We estimated the change in concentration for each patient using linear regression for measured 2,3,4,7,8-PeCDF concentration, then compared the distribution of changes in concentrations with our previous study.

**Results:**

In patients in whom the blood concentration of 2,3,4,7,8-PeCDF exceeded 50 pg/g lipid, the proportion 8.0 % of patients exhibiting half-lives less than 13.3 years fell compared with our previous study (28.2 %), while the proportion with near infinity half-lives increased.

**Conclusion:**

The prolongation of the half-lives was likely a consequence of age-related factors.

## Introduction

The ‘Yusho incident’ was an episode of food poisoning that occurred in western Japan in 1968. Initial reports indicated that Yusho was caused by polychlorinated biphenyls (PCBs). However, following a number of studies, it is now considered that 2,3,4,7,8-pentachlorodibenzofuran (PeCDF) was the main causative compound of Yusho [4, 19]. The concentrations of dioxins and dioxin-like compounds in the blood of Yusho patients have been measured at annual medical checkups since 2001 [8, 18].

Once ingested, dioxins and dioxin-like compounds are excreted extremely slowly, but given the health implications there has been a great deal of interest in the half-lives of these compounds in humans. In patients with high blood concentrations of dioxin-like compounds, half-lives of 1.1 years have been reported, increasing to 7.2 years in patients with low concentrations [9]. Other estimates of dioxin-like compound half-lives include 8.9 years by Masuda et al. [10], 9.6 years by Ryan et al. [15], and 9.1 years by Iida et al. [6]. Many researchers have reported half-lives of PCBs to be less than 10 or 15 years [14, 16]. We previously examined the half-lives of 2,3,4,7,8-PeCDF, which we consecutively measured from 2001 to 2006 in 326 Yusho patients [11]. Notably, among patients with 2,3,4,7,8-PeCDF blood concentrations in excess of 50 pg/g lipid or higher, we identified two groups—one showing a half-life of around 10 years and the other showing no reduction in 2,3,4,7,8-PeCDF levels over time. This suggests that the latter group of patients maintained high blood levels of 2,3,4,7,8-PeCDF [11]. Since the blood concentrations of 2,3,4,7,8-PeCDF in the two groups are three or more times higher than the general public, it seems unlikely that the extended half-lives can be explained by ingestion from food [2, 11]. We re-examined the distribution of the half-lives of 2,3,4,7,8-PeCDF in 395 Yusho patients between 2002 and 2010.

## Materials and methods

The subjects were 395 patients whose blood concentrations of 2,3,4,7,8-PeCDF had been measured three or more times at annual Yusho medical checkups between 2002 and 2010, i.e., 34–42 years since exposure, and for whom the period from the first to last measurement was over 3 years. One patient was excluded because the blood concentration dropped to one-third the original level in the first year, and it remained at that concentration throughout the study period. Table 1 shows the distribution of the patients by sex and age in 2006, which was the median of the measurement period.

**Table 1:** The distribution of the patients by sex and age

Age	Male	Female	Total
20-29	4	0	4
30-39	16	13	29
40-49	18	22	40
50-59	29	36	65
60-69	43	62	105
70-79	56	63	119
80-89	20	12	32
90-99	0	1	1
Total	186	209	395

For each patient, we performed linear regression analysis with the natural logarithm of 2,3,4,7,8-PeCDF concentration in blood lipids as the dependent variable and the year of measurement as the independent variable, using the following equation:

$$ ln{C}_{it} = {k}_i \bullet t + ln{C}_{i0} $$

where *C*_*it*_ is the concentration for patient *i* and time *t*, *C*_*i0*_ is the concentration for patient *i* and time *0*, and *k*_*i*_ (the slope) is the rate of change in concentration for patient *i*.

## Ethical approval

The study was approved by the Graduate School of Medical Sciences, Kyushu University (references 25–166), and the Nara Medical University School of Medicine (reference 281–2).

## Results

**Fig. 1:**
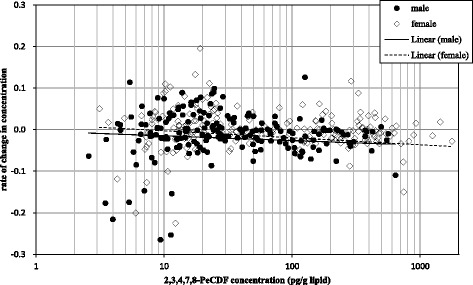
Distribution of rate of change by concentration: *x*-axis, 2,3,4,7,8-PeCDF concentration (pg/g lipid); *y*-axis, rate of change in concentration of 2,3,4,7,8-PeCDF, calculated by linear regression. The black circles represent men; the white open rhombuses represent women. Solid and dashed lines are the linear regression plots of men and women, respectively

Figure 1 is a scatter plot of the 2,3,4,7,8-PeCDF concentrations and rates of change in concentration. Many patients with less than 30 pg/g lipid showed increasing concentrations. This was because the ingestion levels were comparable with the excretion level, and their concentrations were approximately equal to that of the general public. Many patients with over 30 pg/g lipid showed a negative rate of change in concentration, which signified decreasing concentrations. Although the differences were not statistically significant, there was a trend that suggested that the concentration of 2,3,4,7,8-PeCDF falls more rapidly in those patients with higher levels of 2,3,4,7,8-PeCDF.

**Fig. 2:**
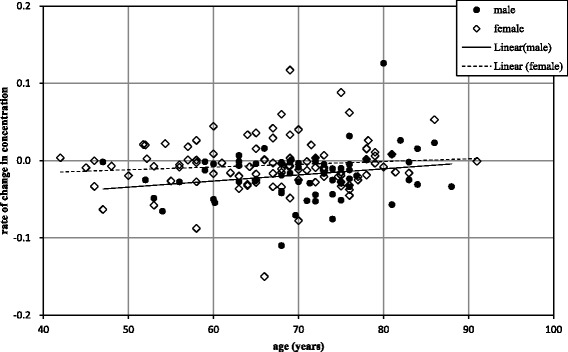
Scatter plot of the ages and rates of change in 2,3,4,7,8-PeCDF concentrations in patients with over 50 pg/g lipid. Solid and dashed lines are the linear regression plots of men and women, respectively. Both lines tend to decrease slowly with increasing age, though the relationship was not statistically significant

Figure 2 is a scatter plot of the ages and rates of change of 2,3,4,7,8-PeCDF concentration in patients with over 50 pg/g lipid. About half women exhibited a positive rate of change, while most men displayed negative rates of change. However, many men aged over 80 years exhibited a positive rate of change in concentration. From these results, it appears that a change in elimination may occur around the age of 80 years in men and 50 years in women; however, the number of patients was too small to confirm this.

**Fig. 3:**
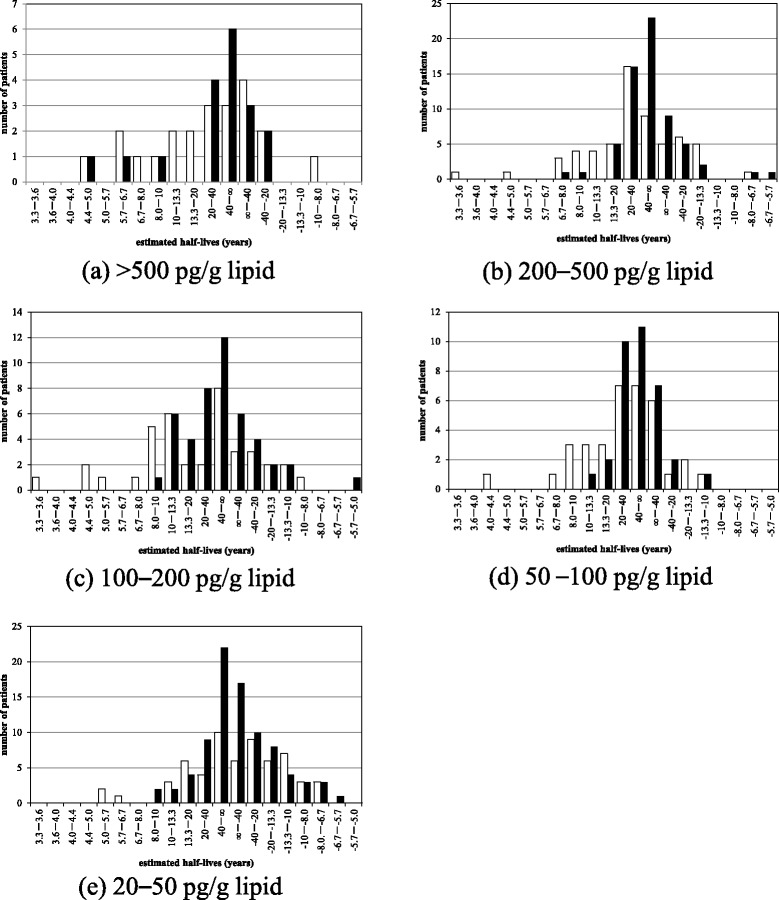
Distribution of half-lives at different concentrations of 2,3,4,7,8-PeCDF compared with previously reported half-lives. White bars indicate the numbers of patients with different half-lives reported by us in 2009 [11]. Black bars indicate the numbers of patients with different half-lives as calculated in this study. (**a**) is the distribution of patients who have concentraion of 2,3,4,7,8PeCDF more than 500 pg/g lipid. (**b**) is the distirubtion for more than 200 pg/g lipid and less thane 500 pg/g lipid. (**c**) is the distiribution for more than 100 pg/g lipid and less than 200 pg/g lipid. (**d**) is the distribution for more than 50 pg/g lipid and less than 100 pg/g lipid. (**e**) is then distribution for more than 20 pg/g lipid and less than 50 pg/g lipid

Figure 3 shows the distribution of the half-lives in the different concentration levels of 2,3,4,7,8-PeCDF, comparing our previous findings (white bars, [11]) with those of this study (black bars). Although the calculated half-lives were variably distributed, we could identify at least two groups: those with half-lives under 10 years and those near infinity. In the groups with 2,3,4,7,8-PeCDF concentrations in blood of >500 pg/g lipid (Fig. 3a) and 200–500 pg/g lipid (Fig. 3b), the proportion of patients with near infinite half-lives, more than 13.3 years, was 96.9 %, a greater proportion than that seen in our previous study (78.3 %). In patients with concentrations of 100–200 pg/g lipid (Fig. 3c), we could identify a group with half-lives of 10.0–13.3 years. In this study, the distribution of half-lives in most patients whose 2,3,4,7,8-PeCDF concentrations ranged between 20 and 50 pg/g lipid (very similar to the general public) appears to be very much like a normal distribution around the infinite (Fig. 3e).

## Discussion

The concentration of dioxins and dioxin-like compounds in the human body is influenced by the balance between excretion and ingestion, change in body weight and measurement error [13, 16]. Current mean blood concentration of 2,3,4,7,8-PeCDF in the general public is 15.2 pg/g lipid (standard deviation ± 8.9 pg/g lipid) [4]. Among healthy individuals, the current concentration is the result of ongoing low-level exposure. This likely explains why Yusho patients with lower 2,3,4,7,8-PeCDF concentration had a near normal distribution of half-lives around the infinite in this study. The half-lives in patients with higher concentrations than the general public are less affected by dietary intake of dioxin-related compounds [2]. For Yusho patients with high 2,3,4,7,8-PeCDF concentrations, such as those in Fig. 3a, the effects of ingestion were negligible. As has been reported previously [11], the half-lives in patients with high 2,3,4,7,8-PeCDF concentrations could be divided into two groups—of around 10 years and near infinity.

Milbrath et al. [13] reported that the concentrations of dioxins and dioxin-like compounds were influenced by changes in body weight. However, our previous study demonstrated that there was no significant relationship between the long-term rate of change in body weight and the half-life of 2,3,4,7,8-PeCDF [12]. Other researchers have reported the half-lives of dioxin-like compounds to be under 10–15 years [14], which is partly consistent with the results of this study; however Ritter and colleagues studied the general public under the assumption that half-lives were equal in different subjects—in other words, that half-lives could be represented by a single value. Their results were straightforward, but ignored individual variations. Aylward et al. [1] studied the half-lives of dioxins in much older people and found them to be under 15 years; however, the median body mass index (BMI) of their subjects was around 32 kg/m^2^, which is much higher than in our study (BMI 23 kg/m^2^); thus, their results may simply not be applicable to the older Japanese population. Grandjean et al. [5] reported half-lives for children; their half-lives were relatively shorter than other reports for adults. In other words, there appear to be shorter half-lives in growing children and longer half-lives in very elderly people. We found that there was a group with half-lives around 10 years and another group with half-lives near infinity. The discrepancy between our results and others might be explained by differences in the compounds measured, as others have reported the blood concentrations of PCBs and PCDDs, the pharmacokinetic behaviors of which are known to be much less extreme than that of 2,3,4,7,8-PeCDF. However, Flesch-Janys studied the half-lives of PCDDs and PCDFs [3], there was no difference the half-lives between PCDDs and PCDFs. The 2,3,4,7,8-PeCDF was the longest half-life among PCDDs and PCDF which were evaluated.

One intriguing finding of this study was the existence of patients with near infinite half-lives of 2,3,4,7,8-PeCDF. The size of the patient group with half-lives around 10 years was smaller than that reported in our previous study [11], whereas the size of the group with near infinite half-lives became larger. Milbrath et al. [13] revealed that menopause resulted in rapid changes in half-lives. Since the subjects in the present study comprised mainly older men and women, menstruation status would not have influenced our results. We have reported that one factor affecting dioxin half-lives is the excretion of dioxin-containing sebum through the skin [12]; this is in accordance with previous findings whereby sebum is an important human excretion pathway [7, 17]. If the excretion of sebum through the skin is decreased by aging, the excretion of dioxins through the skin would likewise diminish.

## Conclusions

The half-lives of 2,3,4,7,8-PeCDF varied among Yusho patients. The half-lives in patients with high 2,3,4,7,8-PeCDF concentrations could be divided into two groups—those of around 10 years, and those near infinity. The former group became smaller in number over time, whereas the latter became larger. The increased number of patients with near infinite half-lives would appear to be explained by age-related factors.
